# Elevation-dependent classification of outdoor thermal stress in a tropical Andean country: a case study of Colombia

**DOI:** 10.1007/s00484-026-03326-x

**Published:** 2026-09-23

**Authors:** Cristian Mejía-Parada, Viviana Mora-Ruiz, Luis Otero-Meneses, Harvey Mieles, Jonathan Soto‑Paz, Brayan A. Parra‑Orobio

**Affiliations:** 1 Barcelona School of Architecture (ETSAB), Universitat Politècnica de Catalunya - BarcelonaTech (UPC), Av. Diagonal 649–651, Barcelona, 08028 Spain; 2 Faculty of Engineering. AVR Research Group, Universidad de Investigación y Desarrollo, Calle 9 # 23-55, Bucaramanga, Santander Colombia; 3 Sustainable Building Design Lab, Dept. UEE, Faculty of Applied Sciences, Université de Liège, Liège, 4000 Liège Belgium; 4 Departamento de Ingeniería Civil: Construcción, Canales y Puertos, E.T.S de Ingenieros de Caminos, Universidad Politécnica de Madrid, c/Profesor Aranguren, s/n, Madrid, 28040 Spain; 5 Facultad de Ingeniería, Grupo de investigación en logística y analítica para una sociedad sostenible-LASSOS, Facultad de Ingeniería, Universidad del Valle, Calle 13 # 100-00, Cali, Colombia; 6 Facultad de Ingeniería Civil, Grupo de Investigación en Ciencias e Ingeniería en Sistemas Ambientales-GCISA-TSEJK, Universidad del Cauca, Popayán, 190002 Colombia

**Keywords:** Outdoor thermal comfort, Thermal stress classification, Polynomial regression, Universal Thermal Climate Index (UTCI), Typical Meteorological Year, Tropical climate, Climate-resilient plannin

## Abstract

Outdoor thermal comfort in tropical mountain countries is shaped by air temperature, humidity, wind, solar radiation and topography, with elevation strongly controlling thermal stress (TS). This study evaluates how elevation modulates outdoor TS in Colombia and proposes an elevation-based thermal stress level (TSL) classification. Typical Meteorological Year (TMY) datasets from the National Solar Radiation Database (NSRDB) were used to compute four thermal indices: Universal Thermal Climate Index (UTCI), Effective Temperature (ETv), Thermo-Hygrometric Index (THI) and the national heat index (IDEAM-HI) for 69,874 locations. For each index, linear and second-degree polynomial regressions between elevation and the annual maximum, mean and minimum values were fitted and assessed using RMSE and R². Polynomial models generally outperformed linear models, particularly for UTCI, explaining up to 96% of the variance in the annual maximum values. The resulting functions were used to derive elevation ranges for different TSL and to produce a TS classification and map. Large lowland areas are predominantly classified within warm and very hot stress conditions, whereas thermally comfortable conditions are mostly restricted to intermediate elevations between 1,080 and 2,050 m.a.s.l. In addition, an exploratory hourly UTCI assessment was performed for four cities using TMY data for representative urban configurations. The urban results qualitatively confirm the elevation-based TS classes and show how diurnal cycles and intra-urban differences modulate TS within each elevation band. The proposed framework provides a climatological, screening-level multi-scale reference for typical outdoor TS in Colombia, enabling baseline spatial prioritization for heat–health risk assessment, climate-resilient planning and comparative assessment planning in tropical Andean regions.

**Supplementary information:**

The online version contains supplementary material available at https://doi.org/10.1007/s00484-026-03326-x.

## Introduction

Thermal stress (TS) strongly affects human health, comfort, productivity, and quality of life. It constrains outdoor work, recreation and mobility and can exacerbate pre-existing diseases, particularly during heat waves (Roshan et al. 2019). In tropical regions, the combination of high air temperature and humidity further amplifies heat stress, posing risks for vulnerable populations and outdoor workers (Sen and Nag 2019; Balogun and Daramola 2019).

In mountain regions the altitudinal gradient adds another layer of variability. Elevation modulates air temperature, humidity and atmospheric pressure, thereby altering the human energy balance and thermal perception (Błażejczyk et al. 2021). In countries with steep topography such as Colombia, large elevation differences occur over relatively short distances, creating pronounced contrasts in outdoor TS and complex microclimatic patterns driven by orography and wind flow (Coccolo et al. 2016; Mejía-Parada et al. 2024b). Understanding how TS changes with elevation is therefore essential for climate-sensitive planning and adaptation (Rangwala and Miller 2012).

Multiple indices have been proposed to quantify TS, including simple thermo-hygrometric metrics and more advanced heat-budget models such as UTCI (Fiala et al. 2012; Jendritzky et al. 2012; Cetin 2020). However, their original stress thresholds were mostly derived for mid-latitude climates with marked seasons. Several studies in tropical and subtropical regions have shown that these generic thresholds may misrepresent local thermal perception and risk, requiring context-specific recalibration (Ahmadi and Ahmadi 2017; Balogun and Daramola 2019; Daemei et al. 2019; de Arêa Leão Borges et al. 2020). In equatorial climates, where seasonal temperature variations are small, but humidity is persistently high, people often adapt to warm conditions and report acceptable comfort at higher index values than those used in standard classifications (Villadiego and Velay-Dabat 2014).

Assessing TS in outdoor environments is a complex process involving various climatic, physiological, and geographical parameters, all influencing the perception of TS (Balogun and Daramola 2019; de Arêa Leão Borges et al. 2020; Li and Liu 2020). Bioclimatic patterns and their effects on the impact of heat stress have been extensively studied, highlighting their influence on human physical and mental health and work productivity (Liu et al. 2019; Hancock 2020). TS can cause serious health problems, such as heat stroke, cardiovascular disease, and dehydration, as well as increasing levels of stress and anxiety (Wilson and Crandall 2011). At the environmental level, thermal stress affects biodiversity, livestock, agricultural yields, and water resources, contributing to droughts and reducing productivity and quality (Waqas et al. 2021; Bilal et al. 2021). It can also accelerate infrastructure degradation and increase energy consumption (Attia and Carlucci 2015; Lai et al. 2019).

This has resulted in the definition of numerous TS indices (e.g., WBGT, ETv, THI, UTCI, PET, SET, PMV) that describe heat stress depending on different thermal, geographic, and physiological conditions (Jendritzky et al. 2012; Coccolo et al. 2016; Walther and Goestchel 2018; Sen and Nag 2019). Each of these indices has its own theoretical and methodological basis for evaluating thermal comfort, considering different combinations of meteorological variables. Often the evaluation of the thermal index offers a versatile and robust tool for understanding and managing TS in various applications, improving quality of life and resilience against adverse climatic conditions (Zengin et al. 2010; Cetin 2019; Liu et al. 2019; Zeren Cetin and Sevik 2020).

For each thermal index, there is a classification based on the level of thermal stress, where the definition of these ranges is crucial to assess and define the TS (Blazejczyk et al. 2012; Zhao et al. 2021; Bozdogan Sert et al. 2021). In regions with marked seasons, thermal amplitudes throughout the year are considerable, resulting in varying thresholds from − 40 °C to + 50 °C. Most thermal indices have been established in climates with seasons, predominantly in the northern hemisphere (Blazejczyk et al. 2012; Zhao et al. 2021). However, thermal variations are minor in tropical areas near the equator, and climates are more stable in spatiotemporal analysis. In these contexts, the ranges of heat stress perception have been adapted to the local environment by different authors (Sen and Nag 2019; Balogun and Daramola 2019; de Arêa Leão Borges et al. 2020). People in these regions usually experience a specific adaptation that makes them perceive thermal stress differently (Villadiego and Velay-Dabat 2014), requiring the modification of thermal index ranges to adequately reflect these areas’ bioclimatic reality.

In specific studies of tropical regions with an altitudinal gradient, it has been observed that altitude affects both the magnitude and frequency of TS. For example, research in the Andes and other tropical mountain ranges has shown that higher-altitude areas experience lower levels of TS (Natarajan et al. 2015) compared to low-altitude areas (Rodriguez-Potes and Villadiego 2020). Mountain barriers force air masses to rise, increasing their density, lowering their temperature, and increasing the environment’s relative humidity. This causes the formation of clouds and alters precipitation in the affected regions (Błażejczyk et al. 2021).

In Colombia, a recent scoping review made by (Medina et al. 2021) shows that most thermal comfort research has focused on indoor environments, particularly in housing and offices located in the Andean region (Natarajan et al. 2015; García et al. 2019; Rodriguez-Potes and Villadiego 2020; Garay et al. 2022). Only a few studies have examined outdoor TS, mainly for coastal cities under hot–humid climates (Villadiego and Velay-Dabat 2014; Rodriguez-Potes and Villadiego 2020). As a result, there is still no country-wide, elevation-based classification of outdoor TS that integrates multiple indices and explicitly accounts for Colombia’s complex topography.

Recent reviews also indicate that elevation-dependent warming is emerging as a key signal of climate change in mountain regions, with enhanced temperature trends at higher altitudes in several mountain systems, including the Tropical Andes (You et al. 2020; Toledo et al. 2021). This reinforces the need to understand how thermal stress responds to elevation in topographically complex countries such as Colombia.

Despite growing interest in tropical heat-stress assessment, previous elevation–thermal stress studies in tropical regions remain insufficient for nationwide decision support in complex mountain countries. First, many contributions are city-scale or site-scale and do not resolve national elevation gradients or transitions across thermal floors. Second, most classifications rely on a single index and do not address cross-index consistency of stress categories. Third, gridded or model-based products often operate at spatial resolutions that smooth sharp topographic gradients, limiting interpretability for mountain valleys and urbanized highlands. Consequently, there is still a lack of nationwide, elevation-explicit outdoor thermal-stress classification frameworks for tropical Andean settings.

This study addresses that gap by evaluating how elevation modulates outdoor TS in Colombia using four thermal indices: the Universal Thermal Climate Index (UTCI), Effective Temperature (ETv), Thermo-Hygrometric Index (THI) and the national IDEAM Heat Index (IDEAM-HI). Typical Meteorological Year (TMY) datasets from the National Solar Radiation Database (NSRDB) are used to derive annual maximum, mean and minimum values of each index for 69,874 locations. We then (1) fit linear and second-degree polynomial regressions between elevation and each index, (2) develop an elevation-dependent thermal stress level (TSL) classification by combining the four indices, (3) map the resulting TSL across Colombia, and (4) perform and exploratory TS assessment in urban settlements located in different TSL. The resulting framework provides a transferable reference for outdoor TS in tropical mountain countries and supports heat–health risk assessment, outdoor work regulations and climate-resilient spatial planning.

This research is organized into six sections. The first section presents a conceptualization of the thermal indices and the aim of this study. The second section introduces the study area, then the third section details the methodology, including the data collection, thermal indices processing, and correlation between elevation and the TS. Then, the fourth section presents the results and discussion of the study, including the new classification of TSL for Colombia. The fifth section includes the limitations of the study and finally, the last section presented the conclusions and perspectives.

## Study area

Colombia, located in northwestern South America, extends between 12°N–4°S and 68°W–78°W, crossing the equator in its southern portion. Its climate reflects the interaction between latitude and rugged topography that modulates solar radiation, temperature, precipitation and humidity. According to the Köppen–Geiger classification (Fig S1.1-A in Supplementary Material 1), Colombia spans tropical rainforest and monsoon climates in lowland Pacific and Amazon regions and temperature, cold, high-mountain climates in the Andes (Peel et al. 2007; Mejía-Parada et al. 2024b). The Andean range crosses the country from south to north, splits into three branches and generates strong elevation gradients and a sequence of traditional “thermal floors” (Fig.S1.1-B).

Warm and very humid conditions dominate below about 1,000 m.a.s.l., intermediate elevations between roughly 1,000 and 3,000 m.a.s.l. correspond to temperate and cold Andean climates, and above 3,000 m.a.s.l. high-mountain and tundra climates prevail. Most of the Colombia’s 52 million inhabitants are concentrated in a limited number of cities distributed along these elevation bands, however in the Caribbean coast are concentration of population especially due to its importance as tourism and key harbor region. Four of them are examined here as urban case studies: Cartagena, a low-land Caribbean city with a tropical savanna climate, Cali (1,000 m.a.s.l.) in the Cauca Valley, Medellín in the Aburrá Valley (1,500 m.a.s.l.), and the Bogotá–Soacha metropolitan area (2,500–2,700 m.a.s.l.) on a high Andean plateau (Medina et al. 2021; Mejía-Parada et al. 2024a). These cities represent the main TS regimes captured by the elevation-based classification developed in this study.

## Materials and methods

**Fig. 1:**
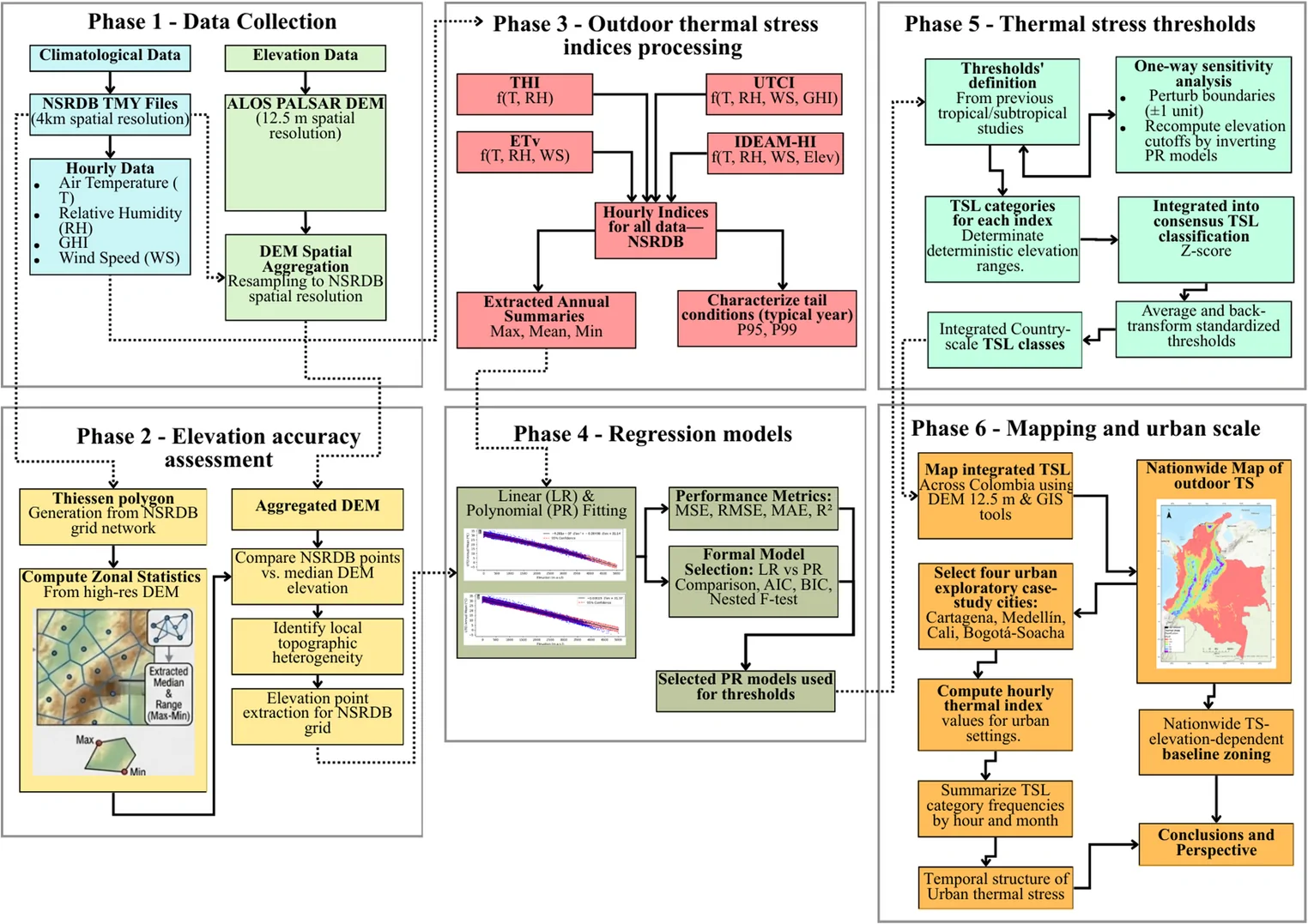
Methodology framework of the present study

The study followed six main phases to derive an elevation-based classification of outdoor TLS for Colombia and to explore its application in selected urban settings. First, climatological and geographical data were compiled. Second, the meteorological variables required to compute thermal indices were extracted and screened. Third, four outdoor thermal indices were calculated at each location. Fourth, linear and second-degree polynomial regressions were fitted between elevation and the annual maximum, mean, and minimum values of each index. Fifth, index-specific elevation thresholds corresponding to predefined TSL categories were derived and then integrated into a consensus classification using Z-score normalization. Finally, the resulting TSL classes were mapped across Colombia using GIS tools, and an exploratory indices analysis was conducted for four representative urban case-study cities. The complete workflow is summarized in Fig. 1.

### Data collection

Climatological data were obtained from the National Solar Radiation Database (NSRDB), which provides Typical Meteorological Year (TMY) files derived from satellite observations(Sengupta et al. 2018). A TMY is a synthetic one-year hourly time series designed to reproduce long-term typical conditions compiled from multi-year records, but it does not reproduce year-specific extremes. In tropical mountain regions, interannual variability and extreme events can be substantial; therefore, the TMY-based results presented should be interpreted as representative baseline thermal-stress regimes rather than as an operational characterization of extreme-event severity or frequency (Siu and Liao 2020; Li et al. 2021, 2023). For Colombia, 69,874 NSRDB grid points were extracted from the 1999–2021 NSRDB record at the native 4 km resolution. At each grid point, hourly air temperature, relative humidity, global horizontal irradiance and wind speed were retrieved as inputs for the thermal-index computation.

Because the NSRDB TMY products are distributed as quality-controlled datasets, no additional corrective gap-filling or data-cleaning procedures were required for the extracted Colombian files. Processing therefore focused on extracting the hourly variables required for each thermal index and preserving consistency throughout the calculation workflow prior to deriving the annual summaries. The hourly records were retained to derive the annual maximum, mean, and minimum values of each thermal index at each NSRDB location.

Topographic information was obtained from the ALOS PALSAR Digital Elevation Model (DEM), with a nominal sparial resolution of 12.5 m. (https://search.asf.alaska.edu/#), Elevation was assigned to each NSRDB grid point after resampling the DEM to the NSRDB support using averaged spatial aggregation.

### Elevation accuracy assessment

Because the DEM and meteorological data differ in spatial resolution, an additional elevation consistency assessment was performed before deriving the country-scale classification. The12.5 DEM was first aggregate to the effective spatial resolution of the NSRDB grid. Thiessen polygons were then generated from NSRDB grid network, and computed zonal statistics were computed from the DEM for each polygon. The NSRDB points was compared with the median DEM elevation within the corresponding polygon, and agreement was summarized using the coefficient of determination (R^2^), mean absolute error (MAE), and root mean square error (RMSE). In addition, sub-gird topographic heterogeneity was characterized through the elevation range (max-min) within each polygon. This procedure was used to evaluate the consistency been the point-based elevation assigned by NSRDB and the aggregated DEM, and to identify where local topographic variability may reduce representativeness in complex terrain.

### Thermal indices selection and processing

Four indices commonly used in outdoor thermal comfort studies were selected because the combine different levels of biophysical complexity and are relevant for both international and national applications: he Universal Thermal Climate Index (UTCI), Effective Temperature (ETv), Thermo-Hygrometric Index (THI) and the IDEAM Heat Index (IDEAM-HI). UTCI is a multi-node heat-budget model widely applied in bioclimatic mapping and heat-health warning systems (Błażejczyk et al. 2021; Philippopoulos et al. 2023). Is important to mention that UTCI was computed using the standard operational procedure, assuming an adult walking at 4 km h⁻¹ (2.3 MET ≈ 135 W m⁻²) and an adaptive clothing model, in which clothing insulation varies from approximately 0.4 to 2.6 clo depending on air(Bröde et al. 2012; Fiala et al. 2012; Jendritzky et al. 2012; Di Napoli et al. 2018). ETv and THI are simpler thermo-hygrometric indices frequently used in temperate, mid-east region and tropical studies (Roshan et al. 2019; Sen and Nag 2019; Balogun and Daramola 2019; Cetin 2020). IDEAM-HI is a national heat index tailored to Colombian climatology and routinely used by the national meteorological service (González 1998). The mathematical expressions (Eqs. 1–6) and implementation details of the four indices are summarized in Table S1.1 on the Supplementary Material 1.

Hourly values of each index were computed at all NSRDB points. Because of the computational complexity of UCTI model, calculations were performed using the validated ThermoFeel (Brimicombe et al. 2022) and PyThermalComfort packages (Tartarini and Schiavon 2020). For the elevation-based analysis, the annual maximum, mean and minimum values of each index were extracted for every location and related to the corresponding site elevation. It is important to note that the thermal indices usually have units in degrees Celsius, except for IDEAM HI, which is considered dimensionless on an inverse scale to the other indices.

To complement the annual summaries and characterize elevated stress conditions within the TMY framework, upper-tail percentiles were also computed from the hourly distributions. For UTCI, ETv, and THI, the 95th and 99th percentiles (P95 and P99) were calculated. For IDEAM-HI, which operates on an inverse hot-stress scale, the analogous hot-tail metrics were represented by P05 and P01. These metrics were used only to characterize the upper or lower tails within the typical year and were not interpreted as interannual extreme-event indicators.

### Regression models between elevation and thermal indices

To quantify how outdoor TS varies with altitude, two regression models were fitted for each index and response variable (annual maximum, annual mean, annual minimum): (i) a simple linear model (LR) and (ii) a second-degree polynomial model (PR). The PR model was adopted as the simplest extension of a linear lapse-rate-type relationship capable of capturing curvature across broad altitudinal gradients in tropical mountains, where near-surface temperature, humidity, radiation, and boundary-layer conditions may vary non-linearly with elevation. In total, 24 models were estimated using elevation as the sole predictor in order to preserve a transparent, transferable first-order classification (thermal floor) at country scale.

Model performance was summarized using R², mean square error (MSE), RMSE and MAE, computed in Python and 95% confidence intervals were obtained for all regressions. To provide formal basis model selection beyond descriptive fit statistics, LR and PR were additionally compared the Akaike Information Criterion (AIC), the Bayesian Information Criterion (BIC), and nested-model F-test (Akaike 1974; GS 1978; Burnham and Anderson 2002). These statistics were used to evaluate whether the inclusion of a quadratic term improved fir sufficiently to justify the additional model complexity. The selected polynomial models were subsequently used to derive the elevation-dependent thresholds of the TLS classification.

### Stress thresholds definition

For each thermal comfort index, different categories have been established to indicate the TSL that humans may experience (Ahmadi and Ahmadi 2017; Roshan et al. 2019; Balogun and Daramola 2019; Daemei et al. 2019). However, as previously mentioned, other studies have shown that in equatorial zones, this categorization is inadequate for describing TS situations. This is because the original categorizations are based on seasonal climates with extreme weather events (winter and summer). In contrast, thermal changes in the equatorial zone are more minor and constant throughout the year, with exceptionally high levels of heat stress.

In this case study, the region has a predominantly tropical climate due to its proximity to the equator; however, the influence of the Andes Mountains ranges that crosses from the south to the center of the country (Fig. 1-B). For each index, TS levels were defined using seven categories ranging from Very Cold Stress (VCS) to Very Hot Stress (VHS). Thresholds were adapted from previous studies in tropical and subtropical climates and from the IDEAM-HI classification proposed for Colombia (González 1998; Sen and Nag 2019; Balogun and Daramola 2019; de Arêa Leão Borges et al. 2020). Table 1 summarizes the thresholds adopted for the study region.

**Table 1:** Defined Index thresholds for the study region

TSL	IDEAM-HI (a/a)	THI (°C)	UTCI (°C)	ETv (°C)
Very Cold Stress (VCS)	> 15	< 5	< 9.1	< 1
Cold Stress (CDS)	13–15	5–13	9.1–14	1–9
Cool Stress (CLS)	11–13	13–15	14–17.4	9–17
Thermal Comfort (TC)	7–11	15–20	17.4–24.5	17–21
Warm stress (WMS)	5–7	20–26.4	24.5–29.1	21–23
Hot Stress (HTS)	3–5	26.4–30	29.1–37.7	23–27
Very Hot Stress (VHS)	0–3	> 30	> 37.7	> 27

To obtain a single, statistically consistent elevation-based classification, the elevation limits derived

Since these thresholds were adapted from prior tropical and subtropical literature rather, a one-way sensitivity analysis was also performed. Thresholds boundaries of Table 1 were perturbed by ± 1 °C for temperature-based indices (UTCI, ETv, and THI) and by ± 1 unit for IDEAM-HI. For each perturbation, the corresponding elevation cutoffs were recomputed by inverting the fitted annual-mean regression model for each index. The Thresholds in Table 1 were therefore specified a priori from tropical/subtropical literature and the official IDEAM-HI scheme; they were not tuned to reproduce Colombian elevation belts. The elevation ranges in Table 2 are a deterministic output of the fitted index-elevation relationships for those predefined boundaries.

**Table 2:** Altitude range levels for each outdoor thermal stress level according to the four indices and the integrated TSL by altitude. *Note: Integrated altitude ranges obtained from the combined assessment of the four thermal indices

TSL by Altitude	Altitude / IDEAM-HI m.a.s.l.	Altitude / THI m.a.s.l.	Altitude / UTCI m.a.s.l.	Altitude / ETv m.a.s.l.	TLS Integrated classification* m.a.s.l.
Very Cold Stress (VCS)	> 3030.5	> 4100	> 3419.7	> 3657.3	> 3552
Cold Stress (CDS)	2610–3030.5	2613.7–4100	2780–3419.7	2685.8–3657.3	2672–3552
Cool Stress (CLS)	2141.5–2610	2228.6–2613.7	2300–2780	1535.1–2685.8	2051–2672
Thermal Comfort (TC)	1037.2–2141.5	1230–2228.6	1200–2300	850.5–1535.1	1080–2051
Warm stress (WMS)	330–1037.2	-90–1230	400–1200	460–850.5	275–1080
Hot Stress (HTS)	< 330	< -90	< 400	< 460	< 275

Using the selected regression models, we then determined, for each index, the elevation ranges associated with the annual mean value of each TSL category (Table 2). We focused on annual means because seasonal temperature variations are relatively small in Colombia, whereas heat and cold stress are experienced throughout the year. To integrate integrated the index-specific elevation thresholds into a single country-scale classification, the thresholds were normalized using Z-scores according to Eq. (7).

7 $$\:Z=\:\frac{X-\mu\:}{\sigma\:}$$

Where Z is the standardized elevation threshold, X is the elevation value (m.a.s.l), µ is the mean of the elevation across indices for a given TSL boundary, and $\:\sigma\:$ is the corresponding standard deviation. The standardized thresholds were averaged across indices and back-transformed into elevation values to obtain the integrated lower and upper bounds for each TSL category.

### Mapping and urban case studies

The integrated TSL classes were mapped for Colombia using the ALOS PALSAR DEM. For each grid cell, the corresponding elevation was assigned to one of the TSL categories derived from the integrated classification, producing a nationwide map of outdoor TS. To explore how elevation-based classification translates into urban conditions, four urban case studies were selected as exploratory case studies (Cartagena, Cali, Medellín and Bogotá–Soacha). These cities were chosen to represent different intra-urban elevations and morphologies. Hourly thermal indices values were then computed for these locations, and the frequency of each TSL category was summarized by hour and by month to examine the temporal structure of thermal stress within each urban context.

## Results and discussion

### Regressions models assessment

**Fig. 2:**
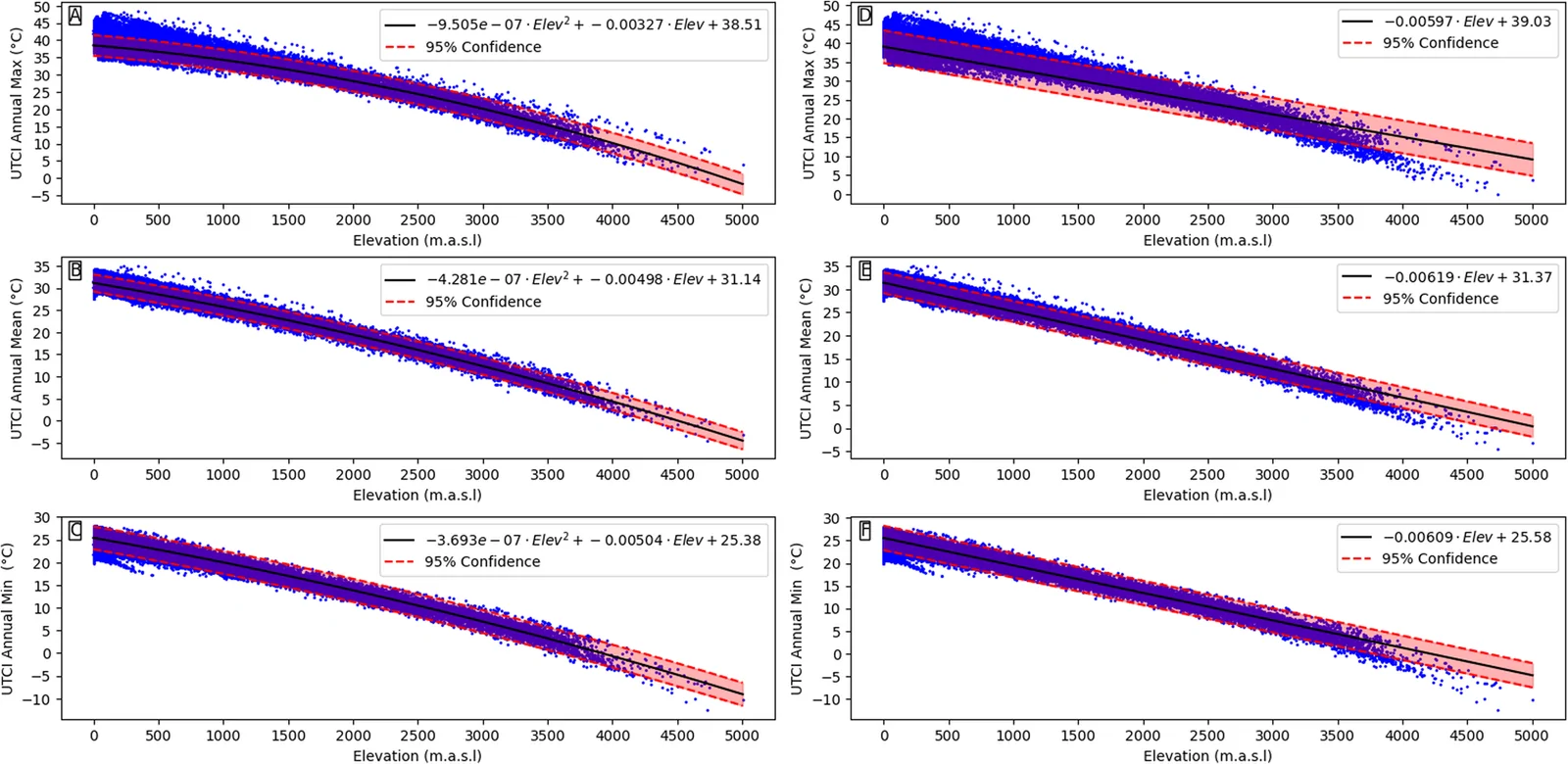
Relation between UTCI and altitude (**A**) UTCI max vs. Altitude polynomial regression; (**B**) UTCI mean vs. Altitude polynomial regression; (**C**) UTCI min vs. Altitude polynomial regression; (**D**) UTCI max vs. Altitude linear regression; (**E**) UTCI mean vs. Altitude linear regression and (**F**) UTCI min vs. Altitude linear regression

In total, 24 regression models (linear and second-degree polynomial) were fitted using elevation as the single predictor for the four indices and for three response variables (annual maximum, mean and minimum). Overall, second-degree polynomial models provided a better description of the elevation–TS relationship than linear models, with lower MSE and RMSE and higher R² values. The improvement was particularly marked for UTCI, for which polynomial models explained up to 96% of the variance in the annual maximum values. Figure 2 illustrates how the polynomial UTCI models capture the non-linear decrease of TS with altitude and reproduce the stronger gradients at low elevations. In the Supplementary Material 1, all 24 indices regression models can be found. This curvature is consistent with the fact that the effective lapse-rate-like behavior of bioclimatic indices can differ between lowlands and high mountains dure to changes in humidity, cloudiness, radiation, and exposure regimes, so a single linear slope may be insufficient across the full national elevation span.

Formal model comparison results support the use of second-degree polynomials beyond the descriptive fit metrics. Across all indices–response combinations, PR models yield lower AIC and BIC than the corresponding linear models (Table S2.2, Supplementary Material 2), indicating that the improvement in fit outweighs the penalty for the additional parameter. Nested-model F-tests also show that the quadratic term provides a statistically significant improvement over the linear formulation, were p-values in most benchmarks models shows a value <1e–16. Importantly, the practical magnitude of the improvement remains index-dependent (Table S2.1): gains are most pronounced for UTCI and ETv, whereas THI shows comparatively smaller differences, consistent with a near-linear first-order response to elevation and the influence of humidity-related variability not captured by altitude alone.

For IDEAM-HI, dispersed behavior was observed, especially in the interval between 2000 and 3000 m.a.s.l. However, polynomial models also improved the fit compared with linear models, although the gains were more modest and overall R² values were lower than for the other indices. This suggests that, despite incorporating some elevation effects in its formulation, IDEAM-HI is more strongly controlled by other climatic variables, which limits its ability to represent TS solely as a function of altitude.

The behavior of ET lies between UTCI and IDEAM-HI. This can be explained due to the similarities between these models, as shown by(Blazejczyk et al. 2012) Comparing the UTCI index against various thermal indices shows that UTCI has a high correlation with ETv. Polynomial models better reproduce the elevation trend at higher altitudes and present narrower confidence intervals, but in some cases linear models achieve slightly lower error metrics due to the concentration of data at intermediate elevations.

For THI, differences between linear and polynomial models are small. Polynomial fits only marginally improve error metrics, and both models achieve similar R² values. This is consistent with the formulation of THI, which emphasizes combined temperature–humidity effects and yields more gradual variations with elevation. In dry and very hot lowland areas, THI predicts high stress levels even at modest elevations, reducing the apparent contrast between lowlands and highlands.

This correlation makes sense, given that previous research in India(Sen and Nag 2019) and Iran(Asghari et al. 2022) has shown that humidity plays a significant role in perceived comfort using this index. However, the high threshold of this index can generate outliers in regions where humidity varies and is not necessarily related to increasing temperature. Furthermore, authors such as(Balogun and Daramola 2019) indicate that THI covers a limited range of climatic conditions and is used more as a guide in regions with limitations in data and processing capacity for more complex indices.

The fit metrics (MSE, RMSE, MAE and R²) for all linear and second-degree polynomial models are summarized in Table S2.1 (Supplementary Material 2). Overall, polynomial models provide a better representation of the elevation–TS relationship, especially for UTCI and IDEAM-HI, where they systematically yield lower errors and higher R² than linear models. This indicates that TS varies with elevation in a non-linear way, a behavior that may not have been captured in previous studies that assumed linear relations between altitude and thermal indices (Gao et al. 2013; Błażejczyk et al. 2021; Krzysztof et al. 2021), often based on sparser weather-station data.

For UTCI, the improvement is particularly clear. In the case of annual maximum values, the polynomial model attains an MSE of 3.35 and R² of 0.96, compared with 6.92 and 0.91 for the linear model. Although the 95% confidence interval of the polynomial fit fails to enclose a fraction of the lowest-elevation points, it tightly constrains the data above 3000 m.a.s.l., indicating a robust performance in high-mountain environments. For mean UTCI, both the error reduction and the narrower confidence band confirm the suitability of the polynomial approximation.

IDEAM-HI also benefits from polynomial fitting, although the contrast with the linear models is smaller. For maximum thermal comfort values, the polynomial model reaches an MSE of 3.04 and R² of 0.83 versus 4.23 and 0.77 for the linear model, yet both remain the lowest R² among all indices. This supports the idea that, in IDEAM-HI, other climatic variables exert a stronger control than elevation alone. ETv shows a mixed behavior: linear models slightly outperform polynomials for annual means, whereas polynomials perform better for minimum values and more closely follow the data envelope, suggesting that the apparent advantage of some linear fits is partly driven by the high concentration of points at intermediate elevations. Finally, THI exhibits very similar performance for both model types (e.g. MSE 2.64 vs. 2.79 and R² 0.90 for maximum values), indicating an almost linear response to elevation and a more dispersed pattern that prevents achieving the levels of fit obtained for UTCI and ETv.

Index-specific patterns are consistent with the underlying constructs of each metric. For UTCI, information criteria and F-tests consistently favor PR, suggesting that the elevation–UTCI relationship exhibits measurable curvature, plausibly reflecting combined non-linear effects of radiation, wind exposure and humidity proxies along complex topographic gradients. For ETv, the strongest preference for PR indicates that elevational changes in evaporative demand and heat exchange regimes are not well captured by a single linear slope, particularly when contrasting lowland and mid-elevation environments. For the IDEAM-HI, the marked improvement under PR is coherent with its formulation as a heat-stress proxy sensitive to humidity, for which the elevation signal can be non-linear due to moisture stratification and regional circulation. In contrast, THI exhibits the smallest separation between LR and PR—especially for annual minimal average—implying that a linear approximation may be practically adequate for some components, even if PR remains favored by parsimony criteria. Overall, these results justify retaining PR as the harmonized functional form for deriving elevation-dependent stress thresholds while acknowledging that curvature strength varies across indices.

Taken together, these results indicate that elevation is a powerful first-order predictor of outdoor TS in Colombia, while acknowledging that other climate factors contribute to dispersion and is not explicitly modelled, particularly when quantified using UTCI. The polynomial models selected for these indices form the basis for deriving the elevation thresholds of the TSL classification, while the more moderate elevation dependence of THI and IDEAM-HI highlights the influence of other climatic factors such as humidity, radiation and wind.

### TSL classification

Once it was determined that the polynomial model was a better fit than the linear model, the elevation ranges for each of the TSL were calculated based on TS thresholds (Table 1). Table 2 shows the elevation ranges according to each of the TSL; however, the level of HTS and VHS was reduced to only Hot Stress (HTS). This is because the established models that evaluate the annual mean require high negative elevation values to satisfy the established ranges.

Due the TS thresholds proposed for the research are adapted from prior studies, we quantified how uncertainty in thresholds values propagates into the derived cutoffs. A one-way sensitivity analysis was performed by perturbing each boundary by ± 1 unit for each index as aforementioned in the methods section, recomputing the corresponding altitude crossings from the annual-mean polynomial regressions, and re-deriving limits using the same consensus procedure as in Table 2. The results of the sensitivity analysis for each index as the integration of all the indices is shown in Tables S2.3–S2.4 in Supplementary Material 2. The results show that individual indices can exhibit shifts on the order of 10^2^ m for a ± 1 unit perturbation, reflection differences in slope/curvature among indices, whereas the integrated classifications are substantially less sensitive: for the key transitions of TSL, the integrated boundaries vary approximately 50–66 m under ± 1 °C. This indicates that the country elevation-based TSL bans are robust to reasonable threshold uncertainty, and that combing indices reduces dependence on any single threshold definition.

To complement the mean-based TSL classification under the TMY framework, we computed upper-tail percentiles from the hourly distributions at all points. Across the integrated TSL classes, P95 and P99 for UTCI, IDEAM-HI, THI and ETv show a consistent separation and ordering (Supplementary Material 2 Fig. S2.1), indicating that the elevation boundaries for the TLS classification capture not only typical mean conditions but also elevated stress levels within the typical-year climatology. These metrics represent the upper/lower tails within TMY and do not substitute interannual extreme-event analyses.

Although Table 1 thresholds are compiled form tropical/subtropical and from the Colombian IDEAM-HI scheme, Colombia still lacks a harmonized nationwide physiological calibration dataset spanning it major climate zones; this limitation has been documented by a recent scoping work on thermal comfort research in the country (Medina et al. 2021).

Because thresholds are defined independently of the derived elevation bands, the subsequent comparison with Colombian literature is used only as an external plausibility assessment and does not constitute validation of thresholds through the same derived ranges. Forn this perspective, available Colombian field evidence nonetheless supports the qualitative plausibility of the adopted stress bands across contrasting climates. Outdoor survey work in hot–humid Barranquilla reports thermal sensation responses under high heat loads and emphasizes strong modulation by radiation, shading and wind exposure, indicating that perceived stress can occur frequently in lowland tropical conditions even with acclimatization (Villadiego and Velay-Dabat 2014; Rodriguez-Potes and Villadiego 2020). In warm climates, adaptive comfort analyses in Cali derive locally informed comfort ranges for public spaces and show that the acceptability window is strongly shaped by ventilation and exposure, consistent with our use of multiple indices and configuration-dependent urban analyses (Vivas and Osuna-Motta 2023). In the high-altitude Andes, Bogotá studies using PET further show the sensitivity of outdoor comfort to urban form and vegetation under climate variability scenarios (Bustamante-Zapata et al. 2022). Finally, recent Colombian outdoor park studies integrating microclimatic measurements and perception surveys in a warm-humid city of Colombia corroborate that subjective discomfort aligns with objective heat-stress proxies in sun-exposed areas, supporting the use of threshold-based categories as a pragmatic baseline when combined with transparency about uncertainty (Rosso-Alvarez et al. 2025). Taken together, these studies support interpreting Table 1 thresholds as a practical nationwide baseline, while motivating future multi-city perception/physiology campaigns to formally recalibrate thresholds for Colombian climates.

Due to the difference in elevation ranges to describe TSL, a statistical adjustment was made to define a TS classification for Colombia, considering all the indices involved. For this, a normalization process of the variables was developed using Z-scores, as described in the methodology. The results can be seen in last column of Table 2, which presents the classification of thermal stress according to topography elevation.

These elevation ranges are broadly consistent with the classical Caldas–Lang climatic classification, which defines warm, temperate, cold and paramo climates in successive elevation belts from 0 to > 3,000 m.a.s.l. (Mejía-Parada et al. 2024b). However, the TS-based classification shifts the thermally comfortable band slightly upward compared with the traditional “temperate” floor (1,000–2,000 m.a.s.l.). This upward shift likely reflects the combined effects of climatic variables not explicitly considered in the Caldas–Lang scheme (e.g. humidity, wind, radiation) and the influence of recent elevation-dependent warming in the Tropical Andes (Rangwala and Miller 2012; You et al. 2020; Toledo et al. 2021).

Recent literature indicates that warming rates in mountain regions can be elevation-dependent, including across the tropical and subtropical Andes, with potentially amplified trends at higher elevations. Such elevation-dependent warming has direct implications for elevation-based thermal-stress classifications: as background temperatures increase, TSL boundaries are expected to shift upslope, potentially compressing cold-stress zones and narrowing the mid-elevation band where thermal comfort is currently most frequent. In practice, highland cities and critical infrastructure located near present-day comfort or cold-stress transitions may experience systematic changes in their baseline regime (e.g., more frequent warm-stress conditions and reduced cold-stress duration). Importantly, the elevation–index regression framework developed here provides a transparent basis for scenario extensions by applying projected temperature changes to the index–elevation relationships and recomputing boundary elevations; however, implementing future scenarios requires multi-year climate projections and explicit treatment of uncertainty, and is therefore left for future work. Because thresholds are defined independently of the derived elevation bands, the subsequent comparison with Colombian literature is used only as an external plausibility assessment, not as validation of the thresholds through the same derived ranges.

The plausibility of the TSL classes is supported by existing comfort studies. Barranquilla, a low-lying Caribbean city, falls within the WMS–HTS bands and field measurements report frequent outdoor thermal discomfort under hot–humid conditions (Villadiego and Velay-Dabat 2014; Rodriguez-Potes and Villadiego 2020). Conversely, Bogotá, at about 2,640 m.a.s.l., lies in the CLS–CDS bands, in agreement with indoor comfort studies that document predominantly slightly cool sensations in naturally ventilated buildings (Natarajan et al. 2015; Rodríguez et al. 2019; García et al. 2019). These correspondences indicate that the elevation-based TSL bands provide a realistic representation of outdoor TS regimes across Colombia.

Region-scale biometeorological mapping studies provide a useful benchmark to contextualize elevation-dependent thermal-stress patterns in tropical and subtropical mountain regions. At continental scale, UTCI-based analyses using gridded reanalysis products reproduce a robust first-order contrast between lowland heat-stress regimes and cooler mountain environments in South America(Miranda et al. 2023), consistent with the elevation-driven structuring observed in Colombia. Such studies increasingly rely on global biometeorological datasets (e.g., ERA-5-derived UTCI products) to characterize the spatiotemporal distribution of thermal stress, but their gridded resolution and reliance on large-scale fields may smooth local topographic effects that are particularly relevant in steep tropical mountains (Di Napoli et al. 2021). Comparable territorial mappings in monsoon-influenced tropical regions with major mountain systems like Southern Asia likewise show strong spatial organization of UTCI classes, with persistently lower UTCI over high-elevation districts and higher thermal stress over lowlands, reinforcing the generality of elevation as a dominant regional classifier of thermal-stress regimes (Kyaw et al. 2023; Thapa et al. 2024).

Within the Tropical Andes, the scarcity of nationwide TS classification products is partly linked to the well-known difficulty of representing climate gradients and extremes over complex terrain. Ecuadorian Andes studies focusing on temperature regionalization and extreme-index evaluation demonstrate that orography strongly structures near-surface climate fields and that gridded products can exhibit limitations in capturing mountain variability, motivating transparent, elevation-aware classification approaches (Fries et al. 2009; Campozano et al. 2017). Complementary Andes-focused comfort research has typically been developed at building-to-regional scales (e.g., high-altitude comfort modeling and adaptation evidence in the Ecuadorian Highlands), highlighting the need for locally contextualized interpretation of thermal stress in high-elevation tropical populations (Miño-Rodríguez et al. 2016). Against this backdrop, our contribution is to provide a nationwide, multi-index, elevation-dependent thermal-stress classification for a tropical mountain country, harmonizing index-specific elevation thresholds into a consensus TSL framework. This type of product supports (i) spatial comparability across climates and elevations, (ii) planning-oriented identification of thermal-stress regimes relevant to public health and infrastructure, and (iii) transferability to scenario-based extensions (e.g., future warming) while remaining transparent about baseline assumptions and threshold uncertainty (Miranda et al. 2023).

### Mapping process and applications

**Fig. 3:**
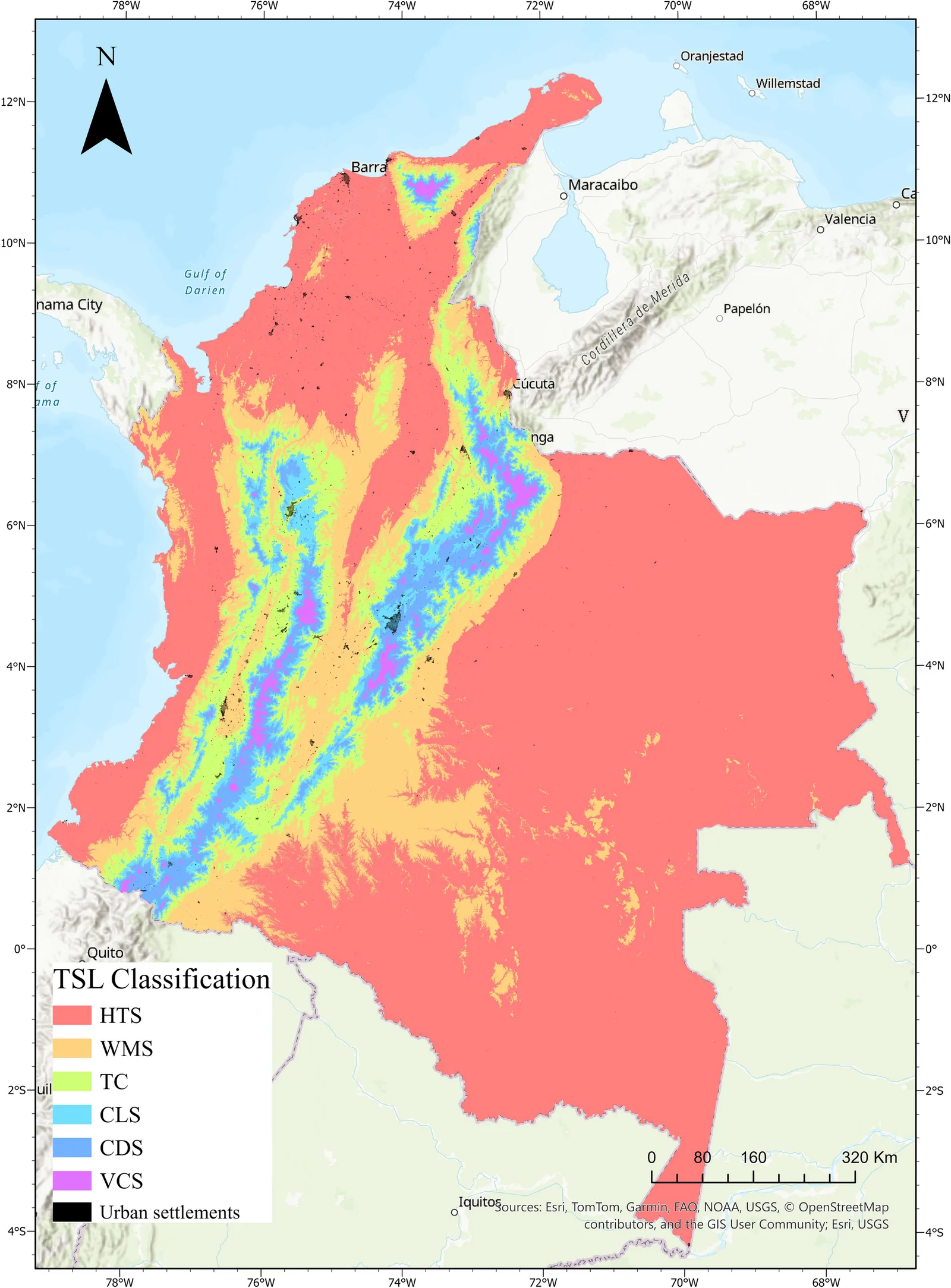
TSL classification mapping for Colombia

The integrated TSL by altitude was applied to the Colombian territory using the ALOS PALSAR DEM (12.5 m per pixel). Because the thermal indices are driven by NSRDB grid cells (~ 4 km) while the mapping uses a 12.5-m DEM, we quantified the elevation-scale consistency using Thiessen polygons at NSRDB scale and DEM zonal statistics per cell. The NSRDB point elevation and the DEM aggregated median elevation showed near-identity agreement (R² = 0.9922), with modest absolute mismatch (MAE = 35.73 m; RMSE = 72.6 m; this analysis is included in the Supplementary Material 4, Fig. S3.5-S3.6). This supports using the high-resolution DEM to spatially render an elevation-based classification while keeping the meteorological forcing scale explicit. However, sub-grid topographic variability can be large in mountainous terrain: the 95th percentile of DEM elevation range within a single 4-km cell reached 1220 m (Supplementary Material 3 Fig.S3.7), indicating that local uncertainty is expected to be higher in the most heterogeneous Andean cells. Consequently, the national map should be interpreted as a regional baseline structured by elevation gradients, with reduced representativeness at sub-cell scales over steep terrain. Each grid cell was assigned to one of the six TSL integrated categories according to its elevation (Table 2), producing a national map of outdoor TS visualized in Fig. 3. The map reveals a clear correspondence between topography and TS regimes: extensive lowland areas in the Caribbean, Pacific and Orinoquia–Amazon regions are dominated by Warm and Hot Stress (WMS–HTS), whereas thermally comfortable conditions occur mainly in intermediate-elevation valleys and plateaus along the Andes. Cool and Cold Stress (CLS–CDS) are restricted to higher Andean slopes and plateaus, while Very Cold Stress is confined to the highest mountain crests above 3,500 m.a.s.l.

In intermediate-elevation regions, bands of TC and WMS follow the foothills of the mountain ranges, giving way to intense heat stress in the surrounding plains. Transitions towards cold climates are less frequent and often coincide with steep terrain where human settlement is sparse. Many medium and small towns in northern Colombia and in inter-Andean valleys are located within WMS–HTS zones, indicating potential exposure of sizeable populations to chronic heat stress conditions.

**Fig. 4:**
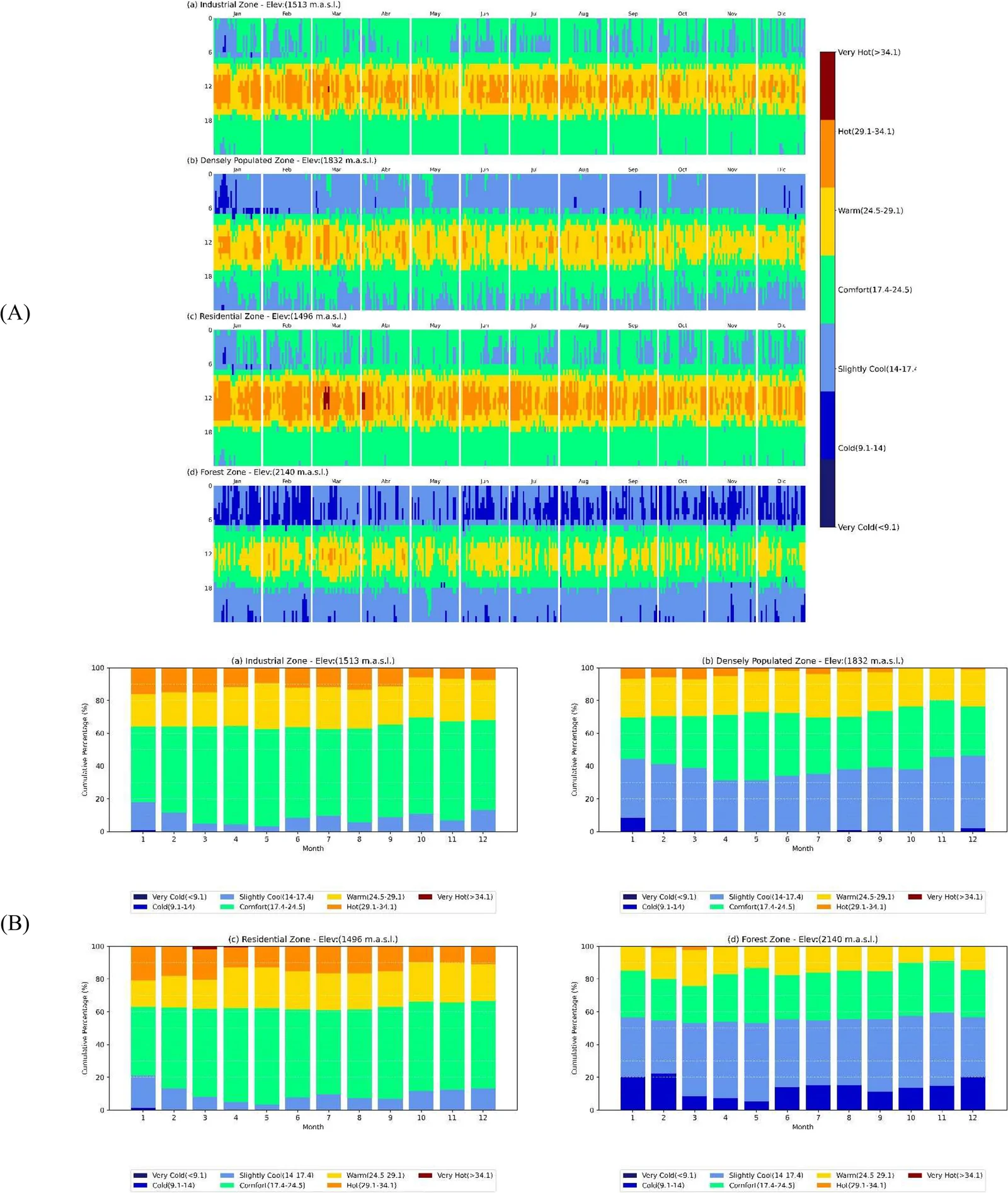
UTCI outdoor thermal stress four different configurations in Medellín City. (**A**) Hourly-daily thermal stress, (**B)** Monthly percentage thermal stress

Major cities, represented by larger markers in Fig. 4, frequently lie close to TSL boundaries, reflecting the strong influence of local topography on urban microclimates. This pattern is consistent with previous work highlighting the role of geographic context, urban morphology and land cover in shaping TS and bioclimatic comfort (Cetin 2019, 2020; Zeren Cetin and Sevik 2020; Philippopoulos et al. 2023).

Thermal stress (TS) has marked social, economic and environmental consequences. In health terms, Wilson and Crandall (2011) show that heat stress can raise cardiovascular strain to potentially lethal levels, while Hancock (2020) reports cognitive impairment under TS, especially when personal protective equipment is used—critical for many occupations. Economically, Waqas et al. (2021) and Ahmed-Farid et al. (2021) link heat stress to reduced productivity, higher healthcare costs and losses in climate-sensitive sectors such as agriculture, where extreme conditions can damage crops and lower yields. Environmentally, heat stress particularly under Urban Heat Island conditions threatens biodiversity and ecosystem resilience when vegetation loss and urban densification increase land surface temperature and TSL. For this reason, Díaz-Chávez et al. (2024) argue that local governments should explicitly assess urban thermal stress and implement mitigation measures to protect residents, especially in climate-change hot spots such as northern Colombia.

Adaptation strategies can operate at multiple scales. In agriculture, Bilal et al. (2021) highlight the potential of nutritional additives, such as vitamin supplements, to alleviate crop heat stress. In cities, green infrastructure helps to reduce heat islands and improve outdoor thermal comfort (Lai et al. 2019).

In this context, an elevation-based TSL classification becomes a useful tool to pinpoint vulnerable areas in regions where TS is strongly modulated by the altitudinal gradient and to prioritize mitigation efforts. The regression models and maps developed here are intended as decision-support tools: they provide stakeholders with an integrated view of TS behavior across Colombia and can inform early project stages by indicating expected thermal index ranges at a given elevation. The use of a high-resolution DEM improves the spatial rendering of elevation-defined TLS classes and reduce the dependence on traditional station-based interpolation. However, it does not increase the effective meteorological resolution, which remains constrained by the spatial resolution of the NSRDB support.

### Outdoor thermal stress in different configurations

To illustrate how the elevation-based TSL classification translates into conditions experienced by urban populations, hourly values for all four thermal indices were analyzed for four Colombian cities located at different levels of the thermal stress classification: Cartagena (HTS), Cali (WMS), Medellín (TS) and the Bogotá–Soacha metropolitan area (CLS-CDS). For each city, four TMY grid points representing different urban configurations and intra-urban elevation differences were selected (Table S3.1 in Supplementary Material 3). For these locations, hourly thermal indices values were computed and aggregated into frequency matrices that indicate, for each month and hour, the proportion of time spent in each TSL category. The selected points for each city are presented in the Supplementary Material 3.

Medellín, with an altitude average of 1,500 m.a.s.l classified as thermally comfortable in the national TLS map, Fig. 4-A presents the hourly UTCI-based thermal stress for this city. Most hours fall within the TC or WMS categories, with relatively few occurrences of Hot Stress and Cold Stress. Daytime periods around midday tend to shift towards WMS, especially in compact, densely built-up configurations at lower elevations, whereas the early morning and late evening hours, and higher or more ventilated sites, show a greater frequency of CLS–CDS conditions. In a forested peri-urban location, Cool and Cold Stress dominate throughout much of the year, highlighting the combined cooling effects of elevation and vegetation.

In contrast, the warm coastal city of Cartagena located mostly at sea level shows a predominance of WMS and HTS during most daytime hours, with TC restricted mainly to the early morning and, to a lesser extent, night-time periods (Fig. S4.2 in Supplementary Material 4). This pattern is consistent with field studies in the Caribbean region, where outdoor thermal comfort is rarely achieved in the afternoon under hot–humid conditions (Villadiego and Velay-Dabat 2014; Rodriguez-Potes and Villadiego 2020). In the other hand, Cali located at 1,000 m.a.s.l exhibits an intermediate behavior (Fig. S4.6), its higher elevation and monsoon-type regime allow a larger fraction of hours within TC, particularly during evening and night, but WMS and HTS still dominate at midday in compact urban settings.

Finally, the Bogotá–Soacha metropolitan area located at 2,500–2,700 m.a.s.l, located in the CLS–CDS bands of the national classification, presents a very different profile. Here, TC and CLS prevail for much of the day, while CDS becomes frequent during night-time and early morning, especially in open or highly elevated sites. HTS is rare and mostly limited to a few midday hours during the warmest months. These contrasts among cities and intra-urban configurations confirm that the elevation-based TSL map captures the broad structure of outdoor TS regimes, while local morphology, land cover and exposure modulate the intensity and timing of heat and cold stress experienced by urban residents.

## Limitations and future works

Several limitations should be considered when interpreting these results. First, the analysis is based on TMY datasets, which smooth inter-annual variability and extreme events. Because TMY, does not reproduce the severity of anomalous years, our classification is designed for baseline spatial characterization and planning-oriented comparisons. Quantifying interannual extremes would require multi-year time series, which is outside the scope of the current dataset. The elevation-based classification therefore characterizes mean background TS conditions and upper-tail conditions within the typical year rather than the full spectrum of heat waves, cold spells or compound events.

Second, elevation is used as the only predictor in the regression models; therefore other geographic factors and local control such as distance to the sea, local land cover or urban morphology are not modelled explicitly and reflected in residual variability. Accordingly, potential misclassification is expected to be greater in steep valleys, ridge environments, and other highly heterogeneous mountain settings where local microclimatic controls are not resolved at NSRDB scale.

Third, although qualitatively plausibility was assessed against existing comfort studies in Colombian cities, a systematic validation against in situ measurements and subjective thermal sensation surveys was beyond the scope of this work. This reflects both the scope of the present work and the practical difficulty of obtaining harmonized observational datasets with sufficient variable completeness, temporal consistency, and spatial/elevational representativeness for the selected urban cases. Accordingly, the proposed classification should be interpreted as a pragmatic national baseline. At the same time, one of the strengths of this framework is its transferability: because it relies on openly available gridded datasets, it can be reproduced in regions with sparse or incomplete station networks, where first-order thermal-stress screening products are still needed. In addition, future changes in elevation-dependent warming patterns under climate change may modify the elevation bands associated with specific TS levels over the coming decades (You et al. 2020; Toledo et al. 2021; Isaak and Luce 2023). Future work should therefore prioritize targeted external validation, local recalibration using high-quality multi-variable station records and physiological surveys, scenario-based analyses of how warming may shift TSL boundaries in tropical mountain regions.

## Conclusions and perspectives

This study examined how elevation modulates outdoor thermal stress in a tropical mountain region using four thermal indices (UTCI, ETv, THI, IDEAM-HI) derived from satellite-based TMY datasets. Elevation was found to be a strong predictor of TS, particularly for UTCI and ETv, for which second-degree polynomial models reproduced up to 96% of the variance in annual maximum values. These models were used to derive altitude ranges for different TS levels and to construct an elevation-based TSL classification.

The integrated classification indicates that large lowland areas in the Caribbean, Pacific and Orinoquia–Amazon regions are predominantly classified within warm and hot stress categories, while thermally comfortable conditions are largely restricted to intermediate elevations between about 1,080 and 2,051 m.a.s.l. Higher Andean slopes and crests are characterized by cool and cold stress. The resulting elevation bands broadly coincide with traditional Colombian “thermal floors”, but the comfort band is shifted slightly upwards, which may reflect the influence of additional climatic variables and recent elevation-dependent warming. A nationwide TSL map derived from the classification highlights that many urban settlements, particularly in lowland regions, are in zones of persistent heat stress.

The exploratory TS assessment for Cartagena, Cali, Medellín and Bogotá–Soacha shows that the national TSL map provides a useful first-order representation of outdoor TS regimes, while local factors such as urban morphology, vegetation and intra-urban elevation differences modulate the timing and intensity of heat and cold stress. These findings support the use of elevation-based TS mapping as a complementary screening-level tool for baseline heat–health risk assessment, occupational heat-risk screening, climate-resilient urban design, and the planning of nature-based solutions and climate shelters in tropical mountain countries. This also makes the framework particularly useful for data-sparse regions, where open available climate products may provide the only feasible basis for this type of first-order thermal-stress screening at territorial scale.

Future research should focus on integrating the elevation-based classification with high-resolution in situ meteorological observations, land-use and socio-demographic data, and physiological and perceptual surveys. Such integration would enable (i) rigorous external validation and local recalibration of stress thresholds, (ii) refined planning-oriented guidance for urban design and occupational heat-risk screening, and (iii) scenario analyses to assess how projected warming may shift TSL boundaries across complex mountains landscapes. The development of calibrated early-warning systems, which requires multi-year observational baselines and explicit treatment of uncertainty, remains a natural extension but is beyond the scope of the present TMY-based framework.

## Supplementary information

Below is the link to the electronic supplementary material.

Supplementary File 1 (DOCX 1.80 MB)

Supplementary File 2 (DOCX 136 KB)

Supplementary File 3 (DOCX 287 MB)

Supplementary File 4 (DOCX 11.6 MB)

## Data Availability

The data will be available by request.

## Abbreviations

DEM: Digital Elevation Model
ETv: Effective Temperature
IDEAM-HI: IDEAM Heat Index
NREL: National Renewable Energy Laboratory
NSRDB: National Solar Radiation Databas
TC: Thermal Comfort
TS: Thermal Stress
TSL: Thermal Stress Levels
THI: Thermo-Hygrometric Index
TMY: Typical Meteorological Year
UTCI: Universal Thermal Comfort Index
