# Supplementary material for: Elevation-dependent classification of outdoor thermal stress in a tropical Andean country: a case study of Colombia
Source: Int J Biometeorol. 2026 Sep 23;70(10):263. doi: 10.1007/s00484-026-03326-x (PMC13601135; doi:10.1007/s00484-026-03326-x)
Supplement: Supplementary file 1 [file 484_2026_3326_MOESM1_ESM.docx]

Elevation-dependent classification of outdoor thermal stress in a tropical Andean country: a case study of Colombia

Cristian Mejía-Parada^1,2*^, Viviana Mora-Ruiz^1^, Luis Otero-Meneses^1^, Harvey Mieles^1^, Jonathan Soto‑Paz^3^, Brayan A. Parra‑Orobio^4^

1 Universidad de Investigación y Desarrollo. Faculty of Engineering. AVR Research Group. Calle 9 # 23-55, Bucaramanga, Santander, Colombia
2 Barcelona School of Architecture (ETSAB), Universitat Politècnica de Catalunya - BarcelonaTech (UPC), Av. Diagonal 649–651, 08028 Barcelona, Spain.
3 Universidad del Valle. Facultad de Ingeniería, Grupo de investigación en logística y analítica para una sociedad sostenible-LASSOS, Facultad de Ingeniería. Calle 13 # 100-00, Cali, Colombia
4 GE&TES Research Group, Department of Environmental and Health Sciences, Popular University of Cesar, Aguachica 205010, Colombia

*****	Correspondence: [cmejia5@udi.edu.co](mailto:shady.attia@uliege.be)

**Supplementary Material 1**

**Table S1.1.** Thermal comfort indices equation implemented in the research.

| **Index** | **Description** | **Model equation** | **Eq.** | **Ref.** |
| --- | --- | --- | --- | --- |
| Discomfort Index (THI) | Evaluated the variate behavior of temperature and relative humidity | $THI=0.8*Ta+\left( \frac{RH*Ta}{500} \right)$  Where, Ta is air temperature (°C), and RH is la relative humidity (%). | (1) | (Balogun and Daramola 2019) |
| Universal thermal comfort index (UTCI) | Includes of climate parameters and comfort variables | $UTCI \left( Ta, Tr,va,RH \right)=Ta+Offset(Ta,Tr,va,RH)$  Where, $Tr$ is the mean radiant temperature (°C), and $va$ is wind speed (m/s) | (2) | (Bröde et al. 2012; Tartarini and Schiavon 2020) |
| Effective temperature index (Et) | Determinates the relative comfort evaluated air temperature, relative humidity, and wind speed | $ETv=$  $37-\frac{37-Ta}{(0.68-0.0014RH+(\frac{1}{1.76+1.40Va*0.75)}}$  $-0.29Ta\left( 1-\frac{0.001RH}{100} \right)$ | (3) | (Sen and Nag 2019) |
| IDEAM-Heat Index | It is a variation of heat index, including the altitudinal gradient. | $IC=\left( 36.5-Ta \right)\left( 0.05+0.04*\sqrt{Va}+\frac{HR}{250} \right)$ ; 0–1000 m.a.s.l  $IC=\left( 34.5-Ta \right)\left( 0.05+0.06*\sqrt{Va}+\frac{HR}{180} \right)$; 1000–2000 m.a.s.l  $IC=\left( 33.5-Ta \right)\left( 0.05+0.18*\sqrt{V}a+\frac{HR}{160} \right)$ ; >3000 m.a.s.l  Where, m.a.s.l. are meters above sea level | (4)  (5)  (6) | (González 1998) |

**Fig S1.1.** (A) Koppen Classification for Colombia (Peel et al. 2007); (B) Digital Model Elevation (DEM).

**Fig S1.2.** Relation between UTCI and altitude (a) UTCI max vs Altitude polynomial regression; (b) UTCI mean vs Altitude polynomial regression; (c) UTCI min vs Altitude polynomial regression; (d) UTCI max vs Altitude linear regression; (e) UTCI mean vs Altitude linear regression and (f) UTCI min vs Altitude linear regression

**Fig S1.3.** Relation between IDEAM HI and altitude (a) IDEAM HI max vs Altitude polynomial regression; (b) IDEAM HI mean vs Altitude polynomial regression; (c) IDEAM HI min vs Altitude polynomial regression; (d) IDEAM HI max vs Altitude linear regression; (e) IDEAM HI mean vs Altitude linear regression and (f) IDEAM HI min vs Altitude linear regression

**Fig S1.4.** Relation between UTCI and altitude (a) UTCI max vs Altitude polynomial regression; (b) UTCI mean vs Altitude polynomial regression; (c) UTCI min vs Altitude polynomial regression; (d) UTCI max vs Altitude linear regression; (e) UTCI mean vs Altitude linear regression and (f) UTCI min vs Altitude linear regression

**Fig S1.5.** Relation between ET and altitude (a) ET max vs Altitude polynomial regression; (b) ET mean vs Altitude polynomial regression; (c) ET min vs Altitude polynomial regression; (d) ET max vs Altitude linear regression; (e) ET mean vs Altitude linear regression and (f) ET min vs Altitude linear regression

**References**

Balogun IA, Daramola MT (2019) The outdoor thermal comfort assessment of different urban configurations within Akure City, Nigeria. Urban Clim 29:. https://doi.org/10.1016/j.uclim.2019.100489

Bröde P, Fiala D, Błażejczyk K, et al (2012) Deriving the operational procedure for the Universal Thermal Climate Index (UTCI). Int J Biometeorol 56:481–494. https://doi.org/10.1007/s00484-011-0454-1

González OC (1998) Metodología para el Calculo del Confort Climático en Colombia. Santa Fe de Bogotá

Peel MC, Finlayson BL, McMahon TA (2007) Updated world map of the Köppen-Geiger climate classification. Hydrol Earth Syst Sci 11:1633–1644. https://doi.org/10.5194/HESS-11-1633-2007

Sen J, Nag PK (2019) Effectiveness of human-thermal indices: Spatio–temporal trend of human warmth in tropical India. Urban Clim 27:351–371

Tartarini F, Schiavon S (2020) pythermalcomfort: A Python package for thermal comfort research. SoftwareX 12:. https://doi.org/10.1016/j.softx.2020.100578
