# Supplementary material for: Elevation-dependent classification of outdoor thermal stress in a tropical Andean country: a case study of Colombia
Source: Int J Biometeorol. 2026 Sep 23;70(10):263. doi: 10.1007/s00484-026-03326-x (PMC13601135; doi:10.1007/s00484-026-03326-x)
Supplement: Supplementary file 2 [file 484_2026_3326_MOESM2_ESM.docx]

Elevation-dependent classification of outdoor thermal stress in a tropical Andean country: a case study of Colombia

Cristian Mejía-Parada^1,2*^, Viviana Mora-Ruiz^1^, Luis Otero-Meneses^1^, Harvey Mieles^1^, Jonathan Soto‑Paz^3^, Brayan A. Parra‑Orobio^4^

1 Universidad de Investigación y Desarrollo. Faculty of Engineering. AVR Research Group. Calle 9 # 23-55, Bucaramanga, Santander, Colombia
2 Barcelona School of Architecture (ETSAB), Universitat Politècnica de Catalunya - BarcelonaTech (UPC), Av. Diagonal 649–651, 08028 Barcelona, Spain.
3 Universidad del Valle. Facultad de Ingeniería, Grupo de investigación en logística y analítica para una sociedad sostenible-LASSOS, Facultad de Ingeniería. Calle 13 # 100-00, Cali, Colombia
4 GE&TES Research Group, Department of Environmental and Health Sciences, Popular University of Cesar, Aguachica 205010, Colombia

*****	Correspondence: [cmejia5@udi.edu.co](mailto:shady.attia@uliege.be)

**Supplementary Material 2**

**Table S2.1.** Fit metrics comparison for each TS model regression

| **Thermal Index** | **Measure** | **Error Type** | **Linear Regression (LR)** | **Polynomial Regression (PR)** | **Δ (LR - PR)** |
| --- | --- | --- | --- | --- | --- |
| UTCI | Annual Max Average | MSE | 6.917 | 3.351 | 3.565 |
|  |  | RMSE | 2.630 | 1.831 | 0.799 |
|  |  | MAE | 2.013 | 1.423 | 0.590 |
|  |  | R^2^ | 0.912 | 0.957 | -0.046 |
|  | Annual Mean | MSE | 1.897 | 1.406 | 0.491 |
|  |  | RMSE | 1.377 | 1.186 | 0.192 |
|  |  | MAE | 1.052 | 0.935 | 0.117 |
|  |  | R^2^ | 0.967 | 0.975 | -0.009 |
|  | Annual Min Average | MSE | 2.679 | 2.365 | 0.315 |
|  |  | RMSE | 1.637 | 1.538 | 0.099 |
|  |  | MAE | 1.293 | 1.254 | 0.039 |
|  |  | R^2^ | 0.948 | 0.954 | -0.006 |
| IDEAM HI | Annual Max Average | MSE | 4.231 | 3.044 | 1.188 |
|  |  | RMSE | 2.057 | 1.745 | 0.312 |
|  |  | MAE | 1.571 | 1.24 | 0.331 |
|  |  | R^2^ | 0.766 | 0.832 | -0.066 |
|  | Annual Mean | MSE | 2.804 | 2.636 | 0.168 |
|  |  | RMSE | 1.675 | 1.624 | 0.051 |
|  |  | MAE | 1.237 | 1.130 | 0.107 |
|  |  | R^2^ | 0.873 | 0.881 | -0.008 |
|  | Annual Min Average | MSE | 2.645 | 2.684 | -0.038 |
|  |  | RMSE | 1.626 | 1.638 | -0.012 |
|  |  | MAE | 1.149 | 1.129 | 0.019 |
|  |  | R^2^ | 0.894 | 0.893 | 0.002 |
| THI | Annual Max Average | MSE | 2.789 | 2.687 | 0.102 |
|  |  | RMSE | 1.670 | 1.639 | 0.031 |
|  |  | MAE | 1.173 | 1.118 | 0.055 |
|  |  | R^2^ | 0.904 | 0.908 | -0.004 |
|  | Annual Mean | MSE | 3.077 | 3.048 | 0.029 |
|  |  | RMSE | 1.754 | 1.746 | 0.008 |
|  |  | MAE | 1.181 | 1.167 | 0.013 |
|  |  | R^2^ | 0.915 | 0.916 | -0.001 |
|  | Annual Min Average | MSE | 3.566 | 3.575 | -0.009 |
|  |  | RMSE | 1.888 | 1.891 | -0.002 |
|  |  | MAE | 1.268 | 1.272 | -0.003 |
|  |  | R^2^ | 0.917 | 0.916 | 0.000 |
| ETv | Annual Max Average | MSE | 3.405 | 3.371 | 0.034 |
|  |  | RMSE | 1.845 | 1.836 | 0.009 |
|  |  | MAE | 1.445 | 1.498 | -0.053 |
|  |  | R^2^ | 0.925 | 0.926 | -0.001 |
|  | Annual Mean | MSE | 3.215 | 3.187 | 0.027 |
|  |  | RMSE | 1.793 | 1.785 | 0.008 |
|  |  | MAE | 1.458 | 1.479 | -0.021 |
|  |  | R^2^ | 0.937 | 0.937 | -0.001 |
|  | Annual Min Average | MSE | 2.881 | 2.621 | 0.260 |
|  |  | RMSE | 1.697 | 1.619 | 0.078 |
|  |  | MAE | 1.376 | 1.342 | 0.034 |
|  |  | R^2^ | 0.951 | 0.955 | -0.004 |

**Table S2.2.** AIC ns BIC fit metrics for each TS model regression

| **Thermal Index** | **Measure** | **AIC LR** | **BIC LR** | **AIC PR** | **BIC PR** | **Δ AIC** | **Δ BIC** | **F-test** | **P-Value** | **Selected model** |
| --- | --- | --- | --- | --- | --- | --- | --- | --- | --- | --- |
| UTCI | Annual Min Average | 220417.70 | 220436.00 | 216837.30 | 216864.80 | 3580.33 | 3571.17 | 3675.587 | <1e-16 | PR |
|  | Annual Max Average | 352859.80 | 352878.10 | 349296.70 | 349324.20 | 3563.03 | 3553.874 | 3657.38 | <1e-16 | PR |
|  | Annual Mean | 216989.30 | 217007.60 | 211881.30 | 211908.70 | 5108.00 | 5098.848 | 5301.27 | <1e-16 | PR |
| IDEAM HI | Annual Min Average | 218645.70 | 218664.00 | 201503.40 | 201530.90 | 17142.30 | 17133.14 | 19429.84 | <1e-16 | PR |
|  | Annual Max Average | 183318.80 | 183337.10 | 181316.10 | 181343.60 | 2002.67 | 1993.519 | 2033.62 | <1e-16 | PR |
|  | Annual Mean | 192570.60 | 192588.90 | 184772.20 | 184799.70 | 7798.36 | 7789.206 | 8252.07 | <1e-16 | PR |
| THI | Annual Min Average | 204384.30 | 204402.60 | 204371.80 | 204399.30 | 12.42 | 3.26 | 14.42 | 1.464e-04 | PR |
|  | Annual Max Average | 225917.90 | 225936.20 | 224202.40 | 224229.80 | 1715.49 | 1706.34 | 1738.70 | <1e-16 | PR |
|  | Annual Mean | 191813.40 | 191831.70 | 191382.60 | 191410.00 | 430.83 | 421.67 | 434.15 | 3.981e-96 | PR |
| ETv | Annual Min Average | 252399.90 | 252418.20 | 244941.00 | 244968.50 | 7458.87 | 7449.72 | 7873.42 | <1e-16 | PR |
|  | Annual Max Average | 236492.50 | 236510.80 | 228639.20 | 228666.70 | 7853.33 | 7844.18 | 8313.55 | <1e-16 | PR |
|  | Annual Mean | 220477.90 | 220496.20 | 211535.60 | 211563.10 | 8942.28 | 8933.12 | 9541.55 | <1e-16 | PR |

**Table S2.3.** Sensibility analysis for TSL-Elevation classification by Index

| Thermal Index | TSL Boundary | Thresholds by Table 2 | Baseline altitude (m) | Altitude–(−Δ) (m) | Altitude–(+Δ) (m) | Shift in altitude (−Δ − baseline) | Shift in altitude (+Δ − baseline) | Altitude domain value (m) | Altitude domain max_ value (m) |
| --- | --- | --- | --- | --- | --- | --- | --- | --- | --- |
| THI | VCS/CDS | 5 | 4097.35 | 4276.949 | 3916.515 | 179.5977 | -180.84 | -2 | 5004 |
| THI | CDS/CLS | 13 | 2613.68 | 2803.967 | 2421.916 | 190.2882 | -191.76 | -2 | 5004 |
| THI | CLS/TC | 15 | 2228.64 | 2421.916 | 2033.824 | 193.273 | -194.82 | -2 | 5004 |
| THI | TC/WMS | 20 | 1238.31 | 1439.706 | 1035.162 | 201.3963 | -203.15 | -2 | 5004 |
| THI | WMS/HTS | 26.4 | Out of range | 119.2855 | Out of range | Out of range | Out of range | -2 | 5004 |
| THI | HTS/VHS | 30 | Out of range | Out of range | Out of range | Out of range | Out of range | -2 | 5004 |
| UTCI | VCS/CDS | 9.1 | 3419.72 | 3545.30 | 3292.40 | 125.5852 | -127.32 | -2 | 5004 |
| UTCI | CDS/CLS | 14 | 2777.87 | 2912.69 | 2640.89 | 134.8231 | -136.97 | -2 | 5004 |
| UTCI | CLS/TC | 17.4 | 2302.74 | 2445.32 | 2157.61 | 142.5792 | -145.13 | -2 | 5004 |
| UTCI | TC/WMS | 24.5 | 1207.71 | 1372.04 | 1039.44 | 164.3281 | -168.27 | -2 | 5004 |
| UTCI | WMS/HTS | 29.1 | 396.074 | 581.28 | 205.18 | 185.2056 | -190.90 | -2 | 5004 |
| UTCI | HTS/VHS | 37.7 | Out of range | Out of range | Out of range | Out of range | Out of range | -2 | 5004 |
| ETv | VCS/CDS | 1 | 3657.28 | 3769.77 | 3543.04 | 112.487 | -114.25 | -2 | 5004 |
| ETv | CDS/CLS | 9 | 2685.79 | 2815.23 | 2553.65 | 129.4361 | -132.14 | -2 | 5004 |
| ETv | CLS/TC | 17 | 1535.06 | 1692.50 | 1372.68 | 157.4373 | -162.38 | -2 | 5004 |
| ETv | TC/WMS | 21 | 850.46 | 1031.02 | 662.320 | 180.5653 | -188.14 | -2 | 5004 |
| ETv | WMS/HTS | 23 | 465.57 | 662.32 | 258.91 | 196.7459 | -206.66 | -2 | 5004 |
| ETv | HTS/VHS | 27 | Out of range | Out of range | Out of range | Out of range | Out of range | -2 | 5004 |
| IDEAM | VCS/CDS | 15 | 3030.53 | 2822.14 | 3231.39 | -208.395 | 200.86 | -2 | 5004 |
| IDEAM | CDS/CLS | 13 | 2605.29 | 2378.87 | 2822.14 | -226.422 | 216.85 | -2 | 5004 |
| IDEAM | CLS/TC | 11 | 2141.48 | 1891.36 | 2378.87 | -250.122 | 237.39 | -2 | 5004 |
| IDEAM | TC/WMS | 7 | 1037.21 | 702.69 | 1342.92 | -334.517 | 305.71 | -2 | 5004 |
| IDEAM | WMS/HTS | 5 | 329.27 | Out of range | 702.692 | Out of range | 373.42 | -2 | 5004 |
| IDEAM | HTS/VHS | 3 | Out of range | Out of range | Out of range | Out of range | Out of range | -2 | 5004 |

**Table S2.4.** Integrated sensibility analysis for TSL-Elevation classification

| TSL Boundary | VCS/CDS | CDS/CLS | CLS/TC | TC/WMS | WMS/HTS | | HTS/VHS | |
| --- | --- | --- | --- | --- | --- | --- | --- | --- |
| Integrated elevation (baseline). μ (m.a.s.l.) | 3551.221 | 2670.657 | 2051.98 | 1083.42 | 396.9738 | Out of range | |  |
| Across-index SD (baseline). σ (m) | 446.4372 | 80.08783 | 350.8605 | 178.7421 | 68.15441 | Out of range | |  |
| No. of indices (baseline) | 4 | 4 | 4 | 4 | 3 | 0 | |  |
| Integrated elevation (−Δ). μ (m.a.s.l.) | 3603.54 | 2727.688 | 2112.772 | 1136.364 | 454.2948 | Out of range | |  |
| Across-index SD (−Δ). σ (m) | 604.1783 | 237.6152 | 379.3923 | 339.9636 | 292.9425 | Out of range | |  |
| No. of indices (−Δ) | 4 | 4 | 4 | 4 | 3 | 0 | |  |
| Integrated elevation (+Δ). μ (m.a.s.l.) | 3495.835 | 2609.65 | 1985.746 | 1019.962 | 388.9274 | Out of range | |  |
| Across-index SD (+Δ). σ (m) | 311.1894 | 167.8361 | 432.9146 | 278.5817 | 273.0536 | Out of range | |  |
| No. of indices (+Δ) | 4 | 4 | 4 | 4 | 3 | 0 | |  |
| Integrated shift (−Δ − baseline) (m) | 52.31883 | 57.03134 | 60.79181 | 52.94311 | 57.32097 | Out of range | |  |
| Integrated shift (+Δ − baseline) (m) | -55.3854 | -61.0068 | -66.2343 | -63.4582 | -8.04641 | Out of range | |  |

**Figure S2.1.** Upper-tail hourly indices metrics within TMY by TSL class (P95/P99). *Note: For IDEAM-HI index the scaled is inverted as mentioned in the manuscript.*
