# Supplementary material for: Elevation-dependent classification of outdoor thermal stress in a tropical Andean country: a case study of Colombia
Source: Int J Biometeorol. 2026 Sep 23;70(10):263. doi: 10.1007/s00484-026-03326-x (PMC13601135; doi:10.1007/s00484-026-03326-x)
Supplement: Supplementary file 3 [file 484_2026_3326_MOESM4_ESM.docx]

Elevation-dependent classification of outdoor thermal stress in a tropical Andean country: a case study of Colombia

Cristian Mejía-Parada^1,2*^, Viviana Mora-Ruiz^1^, Luis Otero-Meneses^1^, Harvey Mieles^1^, Jonathan Soto‑Paz^3^, Brayan A. Parra‑Orobio^4^

1 Universidad de Investigación y Desarrollo. Faculty of Engineering. AVR Research Group. Calle 9 # 23-55, Bucaramanga, Santander, Colombia
2 Barcelona School of Architecture (ETSAB), Universitat Politècnica de Catalunya - BarcelonaTech (UPC), Av. Diagonal 649–651, 08028 Barcelona, Spain.
3 Universidad del Valle. Facultad de Ingeniería, Grupo de investigación en logística y analítica para una sociedad sostenible-LASSOS, Facultad de Ingeniería. Calle 13 # 100-00, Cali, Colombia
4 GE&TES Research Group, Department of Environmental and Health Sciences, Popular University of Cesar, Aguachica 205010, Colombia

*****	Correspondence: [cmejia5@udi.edu.co](mailto:shady.attia@uliege.be)

**Supplementary Material 4**

**Table S4.1.**

**Fig S4.1.** IDEAM-HI Hourly Stress Map for Warm Climate urban settlement– Cartagena City

**Fig S4.2.** UTCI Hourly Stress Map for Warm Climate urban settlement– Cartagena City

**Fig S4.3.** THI Hourly Stress Map for Warm Climate urban settlement– Cartagena City

**Fig S4.4.** Etv Hourly Stress Map for Warm Climate urban settlement– Cartagena City

**Fig S4.5.** IDEAM-HI Hourly Stress Map for Warm-Cool Climate urban settlement– Calí City

**Fig S4.6.** UTCI Hourly Stress Map for Warm-Cool Climate urban settlement– Calí City

**Fig S4.7.** THI Hourly Stress Map for Warm-Cool Climate urban settlement– Calí City

**Fig S4.8.** Etv Hourly Stress Map for Warm-Cool Climate urban settlement– Calí City

**Fig S4.9.** IDEAM-HI Hourly Stress Map for Cool-Cold Climate urban settlement– Medellín City

**Fig S4.10.** UTCI Hourly Stress Map for Cool-Cold Climate urban settlement– Medellín City

**Fig S4.11.** THI Hourly Stress Map for Cool-Cold Climate urban settlement– Medellín City

**Fig S4.12.** ETv Hourly Stress Map for Cool-Cold Climate urban settlement– Medellín City

**Fig S4.13.** IDEAM-HI Hourly Stress Map for Cold Climate urban settlement– Bogotá-Soacha City

**Fig S4.14.** UTCI Hourly Stress Map for Cold Climate urban settlement– Bogotá-Soacha City

**Fig S4.15.** THI Hourly Stress Map for Cold Climate urban settlement– Bogotá-Soacha City

**Fig S4.16.** ETv Hourly Stress Map for Cold Climate urban settlement– Bogotá-Soacha City

**Fig S4.17.** IDEAM-HI monthly frequency assessment for Warm Climate urban settlement– Cartagena City

**Fig S4.18.** UTCI monthly frequency assessment for Warm Climate urban settlement– Cartagena City

**Fig S4.19.** THI monthly frequency assessment for Warm Climate urban settlement– Cartagena City

**Fig S4.20.** Etv monthly frequency assessment for Warm Climate urban settlement– Cartagena City

**Fig S4.21.** IDEAM-HI Hourly monthly frequency assessment for Warm-Cool Climate urban settlement– Calí City

**Fig S4.22.** UTCI monthly frequency assessment for Warm-Cool Climate urban settlement– Calí City

**Fig S4.23.** THI monthly frequency assessment for Warm-Cool Climate urban settlement– Calí City

**Fig S4.24.** Etv monthly frequency assessment for Warm-Cool Climate urban settlement– Calí City

**Fig S4.25.** IDEAM-HI monthly frequency assessment for Cool-Cold Climate urban settlement– Medellín City

**Fig S4.26.** UTCI monthly frequency assessment for Cool-Cold Climate urban settlement– Medellín City

**Fig S4.27.** THI monthly frequency assessment for Cool-Cold Climate urban settlement– Medellín City

**Fig S4.28.** ETv monthly frequency assessment for Cool-Cold Climate urban settlement– Medellín City

**Fig S4.29.** IDEAM-HI monthly frequency assessment for Cold Climate urban settlement– Bogotá-Soacha City

**Fig S4.30.** UTCI monthly frequency assessment for for Cold Climate urban settlement– Bogotá-Soacha City

**Fig S4.31.** THI monthly frequency assessment for for Cold Climate urban settlement– Bogotá-Soacha City

**Fig S4.32.** ETv monthly frequency assessment for Cold Climate urban settlement– Bogotá-Soacha City
