# Supplementary material for: Elevation-dependent classification of outdoor thermal stress in a tropical Andean country: a case study of Colombia
Source: Int J Biometeorol. 2026 Sep 23;70(10):263. doi: 10.1007/s00484-026-03326-x (PMC13601135; doi:10.1007/s00484-026-03326-x)
Supplement: Supplementary file 4 [file 484_2026_3326_MOESM3_ESM.docx]

Elevation-dependent classification of outdoor thermal stress in a tropical Andean country: a case study of Colombia

Cristian Mejía-Parada^1,2*^, Viviana Mora-Ruiz^1^, Luis Otero-Meneses^1^, Harvey Mieles^1^, Jonathan Soto‑Paz^3^, Brayan A. Parra‑Orobio^4^

1 Universidad de Investigación y Desarrollo. Faculty of Engineering. AVR Research Group. Calle 9 # 23-55, Bucaramanga, Santander, Colombia
2 Barcelona School of Architecture (ETSAB), Universitat Politècnica de Catalunya - BarcelonaTech (UPC), Av. Diagonal 649–651, 08028 Barcelona, Spain.
3 Universidad del Valle. Facultad de Ingeniería, Grupo de investigación en logística y analítica para una sociedad sostenible-LASSOS, Facultad de Ingeniería. Calle 13 # 100-00, Cali, Colombia
4 GE&TES Research Group, Department of Environmental and Health Sciences, Popular University of Cesar, Aguachica 205010, Colombia

*****	Correspondence: [cmejia5@udi.edu.co](mailto:shady.attia@uliege.be)

**Supplementary Material 3**

**Table S3.1.** Main characteristics of the four urban case-study cities. Note: the configurations and the elevations change in every city since it depends on the available data

| City | Representative elevation range (m.a.s.l) | Köppen–Geiger climate | Mean annual air temperature (°C) | Role in elevation-based TSL | Urban configuration evaluated (elevation in m.a.s.l.) |
| --- | --- | --- | --- | --- | --- |
| Cartagena | 0–70 | Tropical savanna (Aw) | 27 | Hot stress (HTS) | Industrial Zone (21) |
|  |  |  |  |  | Residential Zone (8) |
|  |  |  |  |  | Rural settlements (8) |
|  |  |  |  |  | Forest zone (63) |
| Calí | 900–1850 | Tropical monsoon (Am) | 24.5 | Warm stress (WMS) | Residential zone -Buildings (1019) |
|  |  |  |  |  | Residential zone – Houses (971) |
|  |  |  |  |  | Rural settlements (1335)  Forest zone (1849) |
|  |  |  |  |  |  |
| Medellín | 1400–2100 | Temperate monsoon (Cwb) | 21.6 | Thermal comfort (TC) | Industrial Zone (1513) |
|  |  |  |  |  | Densely populated zone (1832)  Residential zone (1496) |
|  |  |  |  |  | Rural zone (2081) |
|  |  |  |  |  | Densely populated zone (2551) |
| Bogotá-soacha | 2100–2700 | Temperate oceanic (Cfb) | 13.5 | Cool-Cold stress (CLS-CDS) | Residential zone (2546) |
|  |  |  |  |  | Rural settlements (2589) |
|  |  |  |  |  | Forest zone (2161) |
|  |  |  |  |  |  |

**Fig S3.1.** Urban configuration’s locations for the Bogotá-Soacha City

**Fig S3.2.** Urban configuration’s locations for Cali City

**Fig S3.3.** Urban configuration’s locations for Cartagena City

**Fig S3.4.** Urban configuration’s locations for Medellín City

**Fig S3.5.** Scatter plot between NSRDB elevation and DEM median elevation by each grid of 4 km

**Fig S3.6.** Δ NSRDB and median Elevation (from DEM) histogram by each grid of 4 km

**Fig S3.7.** Location of the Elevation more heterogeneous gird cells (P95)
